# Reducing False-Positive Prediction of Minimotifs with a Genetic Interaction Filter

**DOI:** 10.1371/journal.pone.0032630

**Published:** 2012-03-05

**Authors:** Jerlin C. Merlin, Sanguthevar Rajasekaran, Tian Mi, Martin R. Schiller

**Affiliations:** 1 Department of Computer Science and Engineering, University of Connecticut, Storrs, Connecticut, United States of America; 2 School of Life Sciences, University of Nevada, Las Vegas, Nevada, United States of America; University of Toronto, Canada

## Abstract

**Background:**

Minimotifs are short contiguous peptide sequences in proteins that have known functions. At its simplest level, the minimotif sequence is present in a source protein and has an activity relationship with a target, most of which are proteins. While many scientists routinely investigate new minimotif functions in proteins, the major web-based discovery tools have a high rate of false-positive prediction. Any new approach that reduces false-positives will be of great help to biologists.

**Methods and Findings:**

We have built three filters that use genetic interactions to reduce false-positive minimotif predictions. The basic filter identifies those minimotifs where the source/target protein pairs have a known genetic interaction. The HomoloGene genetic interaction filter extends these predictions to predicted genetic interactions of orthologous proteins and the node-based filter identifies those minimotifs where proteins that have a genetic interaction with the source or target have a genetic interaction. Each filter was evaluated with a test data set containing thousands of true and false-positives. Based on sensitivity and selectivity performance metrics, the basic filter had the best discrimination for true positives, whereas the node-based filter had the highest sensitivity. We have implemented these genetic interaction filters on the Minimotif Miner 2.3 website. The genetic interaction filter is particularly useful for improving predictions of posttranslational modifications such as phosphorylation and proteolytic cleavage sites.

**Conclusions:**

Genetic interaction data sets can be used to reduce false-positive minimotif predictions. Minimotif prediction in known genetic interactions can help to refine the mechanisms behind the functional connection between genes revealed by genetic experimentation and screens.

## Introduction

Minimotifs are short contiguous peptide sequences in proteins that are associated with known biological functions. Minimotifs are generally of less than 15 residues in length and confined to a single secondary structure element. Functions encoded by minimotifs include direct covalent modification of the minimotif, binding determinants for other molecules, and protein trafficking tags.

Minimotifs are defined by a common set of attributes for their sequence and function [1]. A collection of the same type of minimotif in a set of proteins is often reduced to a consensus sequence or position-specific scoring matrix (PSSM). Consensus sequences indicate completely or partially conserved positions, as well as completely redundant positions often indicated by an “x” (e.g., PxxPx[KR] where “x” indicates any amino acid and [KR] indicates either amino acid in the 6^th^ position). PSSMs are matrices that indicate the probability of the 20 amino acids at each position of the minimotif.

Consensus sequences and PSSMs can be used to predict new minimotifs, and thus new functional elements in protein queries. However, because the minimotifs are relatively short when compared to the more complex sequence definitions for protein domains, there is most often a high probability that minimotifs occur in a protein by random chance. Thus false-positive predictions are a general problem in minimotif prediction by websites such as Minimotif Miner, Eukaryotic Linear Motif Server, and ScanSite [2]–[5].

We and other groups have used other types of data to reduce false-positive predictions including protein-protein interactions, molecular and cellular protein functions, evolutionary conservation, protein disorder, protein structure, protein surface prediction, and protein localization [2], [6]–[10]. Although each of these filters reduces false-positive predictions, it remains a problem and new approaches to reduce false-positives are needed.

In this paper, we assessed whether genetic interaction (GI) data can be used to reduce false-positive minimotif predictions, and implemented several filters as a part of the Minimotif Miner web system [8], [2]. Systematic reverse genetic analysis of yeast, worms, flies, and several other organisms provides a rich data set of true-positives with 100,000 s of GIs that can be used to refine minimotif prediction. Using GIs is likely to have value in minimotif prediction because there are some examples where GI of a minimotif in one protein with a target protein that binds or modifies the minimotif is already known. For example, Jnk kinase has a GI with several of its natural substrates [11] and Polo binds a motif in Mtrm and both proteins have a GI [12].

One potential caveat of this approach is that several papers indicate that only a portion of GIs map to physical protein-protein interactions [13]–[15]. We do not see this as a critical problem in minimotif analysis because a portion of minimotifs is not expected to have identifiable physical interactions. For example, an enzyme that catalyzes a covalent change of the minimotif (e.g., lipidation, phosphorylation, proteolysis, etc.) is a typical enzyme-substrate interaction and such transient complexes are most often not detected by high-throughput techniques used to identify protein-protein interaction, but may still have a GI related to the enzyme/substrate relationship.

## Methods

### Data Sources

GIs were derived from several sources as shown in **Table 1**. Databases such as Biological General Repository for Interaction Datasets (BioGRID) database, Flybase, NCBI Entrez-Gene, *Saccharomyces* genome database (SGD) contain information about GIs [16]–[19]. We used this information to reduce the false-positives in the predictions of minimotifs using Minimotif miner by filtering the motifs, based on known GIs. The databases are chosen based on the public availability, reliability, and amount of data. There is a total of ∼700,000 GIs from multiple species in these databases.

**Table 1:** Sources of genetic interaction data.

Data source	Species	interactions tested	# genes	Reference
BioGrid	Many species	124410	9020	[16]
SGD	*Saccharomyces cervesiae*	151046	7155	[17]
Flybase	*Drosophila melanogaster*	76411	2904	[18]
Entrez Gene	Many species	387159	-	[19]

The Minimotif Miner 2.3 data model has information about ∼5300 verified minimotifs along with the source protein where the motif was found, and the target protein that imparts the biological function to the minimotif. This data was used to evaluate the efficiency of several GI-based filtering algorithms in reducing false-positive predictions.

### Implementation

We have installed the GI filters on the Minimotif Miner 2.3 website along with existing filters, such as the protein-protein interaction filter, molecular and cell function filter, etc. This enables the users to build custom filtering methodologies based on their requirements or interest. As with the other filters, we also provide users with the exclude option, to examine the motifs that do not have known GIs.

### Genetic interaction filters

We intended to develop a set of algorithms that uses GI data to refine the predictions of minimotifs in MnM. We devised three variations of GI-based filtering algorithms for evaluation. Since many GIs are conserved among related and diverse species, these interactions can be used to identify those minimotifs that have a previously known genetic relationship. The first basic algorithm (designated as “Genetic Interaction (GI) Filter”) is as follows: Let P be any putative motif, let S be its source protein, and let T be the target protein associated with the motif P. Let S′ be the gene that encodes protein S, and T′ be the gene that encodes protein T. All the databases containing the GIs are searched for any direct interaction between S′ and T′. If an interaction exists, the motif P will be retained by this basic GI filter, otherwise the motif P will be removed by this filter. This is repeated for all of the T proteins that are predicted for a particular S query. Protein and Gene alias names are taken into account while searching the databases in order to enforce a thorough search in the database.

A set of GIs can be used to build GI networks that contain nodes that represent genes and edges that represent interactions. This structure enables us to explore higher order interactions in the network that are not direct. These second-order, third-order, etc., GIs between nodes may be useful for minimotif filtering and our “GI-node Filter” algorithm is based on this concept. Given a putative motif P along with its source protein S and it target protein T, the genes of proteins S and T are located from different sources. Let the genes of S and T be S′ and T′, respectively. The following steps are repeated for N number of times, N being the node count. All the genes interacting with S′ and T′ are identified. Let S″ be the new set of genes identified to be interacting with S′ and T″ be the set of genes identified to be interacting with T′, respectively. Now, the GI databases are searched for the interaction between any genes in the set S″ with a gene in the set T″, and for the interaction between any genes in the set of T″ with a gene in the set S″. If an interaction is found, the motif P is retained by the filter. Otherwise, it proceeds to find the interacting genes of S″ and T″ iteratively based on the node count. If there is no interaction even after Nth iteration, the motif P gets filtered out by the algorithm. The size of the interaction network for a gene grows exponentially as N increases. When we tested this filter with the node count of 2, 3 and 4, the results on nodes 3 and 4 produced very poor selectivity. So, we limited our experiments to a node count of 2.

Minimotifs are often conserved across species and taxa [20]–[21]. In general, a GIs in one species is a poor predictor of a GIs between orthologs and paralogs in other species, however it is possible that many of those GIs that are conserved are mediated through minimotifs. Thus, we assessed if minimotif source/target pairings that have a known GI in one species could be used to extrapolate a valid minimotif in another species. To test this hypothesis, we designed an extension of the filtering algorithm (“GI-HomoloGene Filter”) that aims at assessing the conservation of gene interactions in orthologs and paralogs. For a given pair (S, T) for a putative motif P, S being its source protein and T being its target protein, HomoloGene database is searched for the HomoloGene clusters of S an T. Let S′ be the HomoloGene cluster of S, and let T′ be the HomoloGene cluster of T. Gene interaction databases were used to check if GI (A, B) or (B, A) exists such that A belongs to S′ and B belongs to T′. If one such interaction is found, the motif P passes the filter, else it fails the filter. We also enforced an additional constraint that if there exists an interaction (A, B), then both A and B should belong to the same species for the putative motif P to be retained by the filter.

### ROC and statistical analysis of minimotif filters

ROC (Receiver Operating Characteristic) curves for comparing GI filters were generated using R software package [22]. A ROC curve is a graphical plot of true positive rate against the false positive rate for different filter thresholds. The area under the curve is a measure of the accuracy of the filter, and the p-value specifies the statistical significance of the filter. The calculated binomial curve fit is shown in the figures.

## Results

### Evaluation of genetic interaction filter algorithms

We wanted to evaluate which filters performed best by yielding a clear separation between true positives and false-positives. The effectiveness was tested by comparing metrics of a set of verified motifs to a set of known negatives. Minimotif Miner database 2 was used as the source of data for verified motifs, as it has a total of ∼5300 minimotifs annotated from the literature and has supporting experimental evidence. Each minimotif has associated information such as source protein, which contains the minimotif and a target protein that engages the minimotif, respectively, and is associated with an activity such as binds, modifies, or traffics. About 3000 minimotifs have both source and target accession numbers that can be cross-referenced to GI data. Therefore, the MnM2 database was used as the source for validated motifs for the true dataset.

As there is no direct access to a true-negative dataset of minimotifs, we generated a negative dataset comprised of protein pairs that are not likely to have a minimotif relationship or a genetic interaction. We randomly paired genes for this purpose, since the number of known GIs relative to the total number of possible GIs is negligible. For instance, 25,000 genes have ∼312 million possible pair-wise interactions, but the number of known GIs is small and should not impact our conclusions. We generated ∼27,000 such pairs of source-target genes, and used this as our negative dataset in the process of validating the filters against false-positives.

Measures such as sensitivity and selectivity were employed to validate our algorithms. Our sensitivity analysis measures if a putative motif that is retained by the filter is indeed a part of the true dataset. It is the percentage of true positives that are retained by the filter. Our selectivity metric was based on a computation of the percentage of true-negatives that are accepted by the filter. Thus, algorithms with a higher sensitivity and a lower selectivity are desirable. Hence, the discrimination ratio (DR), the ratio of sensitivity to selectivity, with values more than 1 is favorable. The higher the ratio, the more favorable the filter is in discriminating true minimotifs from incorrect predictions.

The results comparing metrics for the three GI filters are shown in **Table 2**. The basic GI filter performed best recovering ∼21% of the true positives and had a strong preference for retaining positives rather than negatives. As expected, the analysis of the GI-node filter showed a much higher sensitivity, but the selectivity was compromised producing a lower discrimination ratio that the basic GI filter. This was using a distance of 2 GI nodes; analysis of 3 and 4 nodes produced much poorer selectivity (data not shown). Likewise the GI-HomoloGene filter also yielded poorer selectivity and also had the undesirable property that it only had a modest increase in sensitivity over the basic GI filter. We also combined both the GI-node and GI-HomoloGene filters and found that the combined filters were not as effective as the individual filters. Therefore, we conclude that the basic GI filter was the best performing filter on the test dataset.

**Table 2:** Evaluation of genetic interaction filtering algorithms.

Filter	Sensitivity	Selectivity	1DR
GI	21.2%	2.9%	7.3
GI-node	56.2%	12.6%	4.5
GI-HomoloGene	24.3%	11.9%	2

1 . DR = discrimination ratio.

### GI algorithms in combination with other Filters

We wanted to know whether the GI filters were providing any additional information for reducing false positive minimotif predictions when compared to other existing minimotif filters. The frequency filter is based on the minimotif complexity and likelihood of occurrence of a minimotif [2] and the cellular function filter is based on whether or not source/target pairs share a common cellular function [10] derived from the Gene Ontology database [23]. These filters are based on two conceptually different principles than a GI filter.

To determine if the GI filter contains orthogonal information content we compared each filter with various pairwise filter combinations. The GI filter performed significantly better than the frequency score and cellular function filter. The area under the ROC curve (p-values) were 0.93 (p = 2.9*10^−08^) for the GI filter as compared with 0.72 (p = 0.08) for both the frequency score and cellular function filter (0.72, p = 0.12) respectively (**Table 3**) [8], [10]. This indicates that the GI filter contains orthogonal information for reducing minimotif false positives that is not present in either the frequency score or cellular function filter.

**Table 3:** Statistical comparison of the efficacy of different minimotif filters and filter combinations.

Filter	Area under ROC	p-value
Frequency score	0.72	0.08
Cellular function	0.72	0.12
GI	0.93	2.9*10^−08^
PPI	0.97	3.8*10^−07^
GI + Frequency score	0.96	1.1*10^−06^
GI + Cellular function	0.95	1.5*10^−06^
GI + PPI	0.96	1.1*10^−06^

We next investigated if using the GI filter in pairwise combinations with the frequency score or cellular function filter produced better filtering results. The area under the ROC curve was modestly better for these filter combinations (0.95–0.96 when compared with the GI filter (0.93), but the p-values were not as high for the pairwise filter combinations (2.9*10^−8^ vs. 1.1*10^−6^ −1.5*10^−6^). It was also seen that the novel motif prediction rate for GI filter when compared against frequency score filter is 24% and that with the cellular function filter is 56%. Similar results were observed when the GI-HomoloGene filter was used in this analysis (data not shown). Although the pairwise filter results analysis are not as striking, collectively the filter comparisons show that the GI filters contain additional informational content with regard to eliminating false positive minimotif predictions.

We also have investigated the difference between the GI filter and the Protein-Protein Interaction filter. It turned out that in the true dataset 871 motifs passed the Protein-Protein Interaction filter, while 944 passed the either-or combination of GI filter and Protein-Protein Interaction filter. This combination of GI and Protein-Protein Interaction filters resulted in an 8.4% increase in the sensitivity, which indicates that the Genetic Interaction and the Protein-Protein Interaction filters play a complementary role, to a certain degree, in predicting a true minimotif and by using the union of both a better sensitivity can be achieved.

### Do genetic interaction filters work better on different types or properties of minimotifs?

Most minimotifs in the MnM database are for binding or posttranslational modifications. When analyzed separately 56% of the posttranslational modification minimotifs have a known genetic interaction, while only 19% of binding motifs had a known GI. Statistical analysis of this stratification using ROC plots shows that the GI filter for both the binds and modifies minimotifs groups are significant (p<0.01)(**Table 4** **)**.

**Table 4:** ROC curve statistics for differ types of minimotifs.

Minimotif type	Area under ROC	p-value
All	0.93	2.9*10^−08^
Binds	0.95	1.5*10^−06^
Modifies	0.84	4.8*10^−03^
Phosphorylates	0.87	7.0*10^−03^

**Figure 1:**
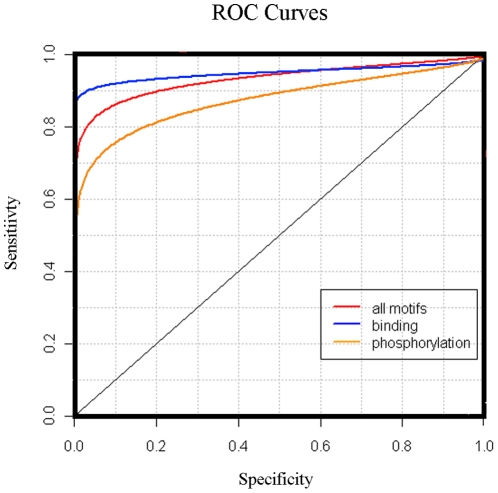
ROC curves for the GI filter with different types of minimotifs. ROC curves are generated using R software package with activity and sub-activity as the underlying variables. The binomial curve fit is shown. The areas under the ROC curves are 0.93 for all minimotifs (red lines), 0.95 for binding motifs (blue lines) and 0.87 for phosphorylation minimotifs (orange lines).

The most common posttranslational modification annotations in the MnM 2 database are phosphorylation sites (n>100) and protease sites (n = 20); 49% and 80% of these motifs had known GIs, whereas 7% and 0% had GIs when the random dataset was analyzed as a control. ROC curve analysis shows that the GI filter for the phosphorylation, as well as the all minimotif and bind minimotif groups are good minimotif filters (p<0.01) (**Figure 1** **,** **Table 4** **)**. We also note that the HomoloGene-GI filter (data not shown) had similar performance to the GI filter. An ROC curve analysis using minimotif length as a variable was performed, but did not produce any discernable pattern (data not shown). Collectively, these analyses suggest that a high percentage of some types of minimotifs have GIs and supports the approach of using GIs as filters for eliminating false positive minimotifs.

### Adapting MnM2.3 User Interface for GI Algorithms

**Figure 2:**
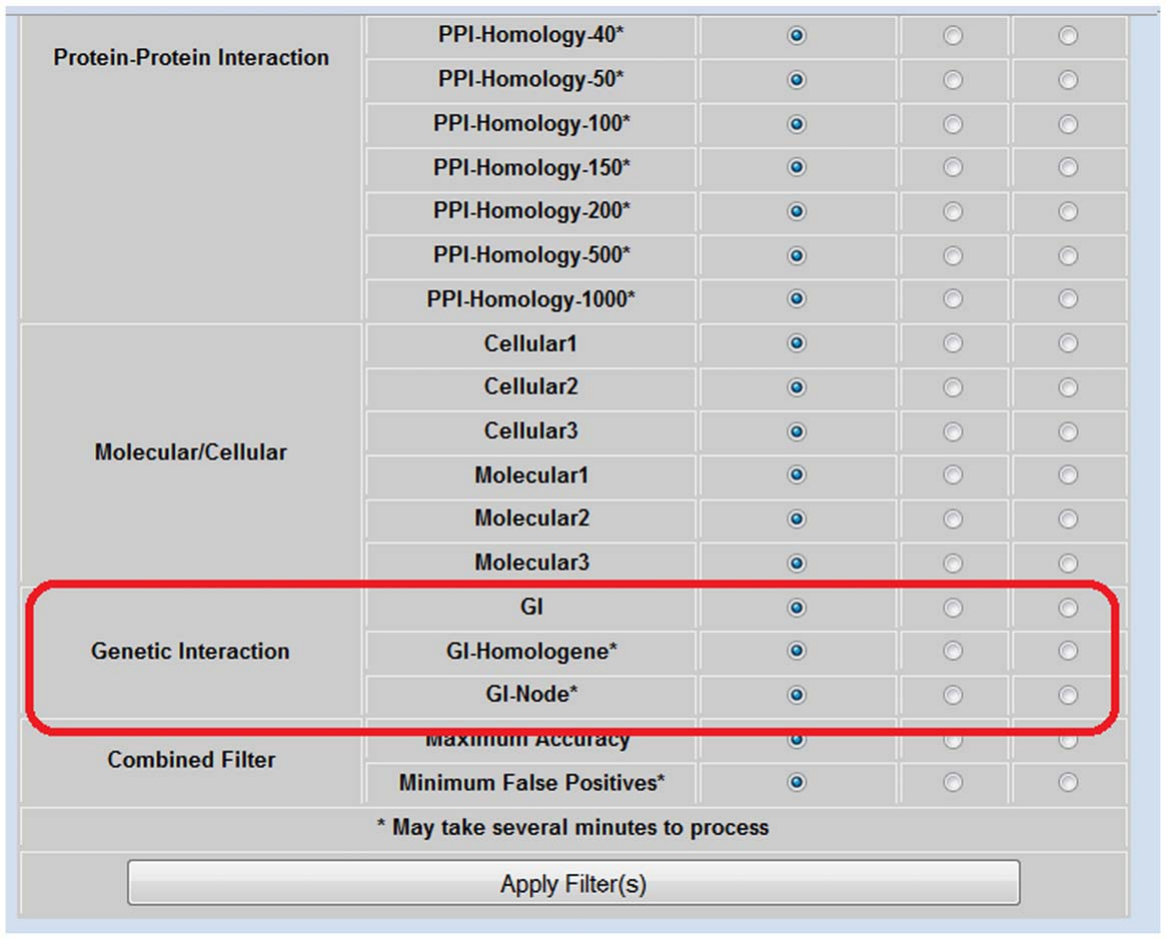
Screen Shot of Minimotif Miner 2.4 filter menu. GI filters were added as part of MnM website 2.4 located under the motif filter pull down section. The options for filtering with ‘GIs’ are outlined with a red box. This filter can be used on its own or in combination with filters. There are also options to check boxes to include or exclude minimotifs with GIs.

To enable the users to access these filters, we have updated the MnM 2.3 user interface to include these filters under the section of GI filters. This contains GI, GI-node and GI-HomoloGene filters (**Figure 2**). These filters can be applied to the resulting list of putative motifs by enabling the check box next to the respective filter. These filters can be used in combination with other filters. If it is preferred not to include the results based on a particular filter, there are options to disable the filter as well. Based on the filters selected, minimotif results table gets updated with the results that are retained by the filters. This enables the users to focus their search by allowing a better control on the selection criteria. The MnM help section has more information regarding filtering.

## Discussion

In this paper we explore the use of GIs as an additional source of data that can be used to help overcome the problem of predicting false-positive minimotifs. We expected that GIs would provide a good basis for a false-positive filter because GIs, like minimotifs reveal functional connections between proteins. The first filter we tested was a basic GI filter where we removed any minimotif where the source/target pair did not correspond with a direct pairwise GI. Evaluation of the basic GI filter using a test dataset revealed good discrimination for retaining true positive minimotifs, while rejecting false-positive minimotifs. This filter performs with similar efficiency to several other filters we have reported, but uses a conceptually different type of data [9]–[10].

We had wanted to expand the utility of this filter to more broadly cover other species since many GIs are discovered in tractable model organisms such as yeast, flies, and worms. We used the HomoGene database to expand any predicted GI across species lines and expanded the basic GI filter to include these predicted GIs. Analysis of the test dataset revealed that this approach was not as robust as the basic GI filter, with a slightly higher rate of true positive predictions, but a much higher rate of false-positive predictions.

The observation that the HomoloGene-GI filter did not significantly improve prediction of minimotifs was mostly consistent with previous observations about GI networks. While it is thought that gene function is conserved among divergent species [24], GI networks are generally though not to be well conserved. Approximately 29% of GIs are conserved among closely related *Saccharomyces* species separated by >100 million years of evolution [13]. Less than 5% of worm interologs (conserved interactions) are conserved in yeast [15].

Another adaptation of the GI filter we tested was to examine if the path length (number of nodes) could be used to improve minimotif predictions. GI networks are composed of pairs of genes in complementary pathways or are involved in the same pathway [25]. Since many minimotifs are regulatory, minimotifs may provide feedback by connecting nodes in pathways that are more than one node away. This hypothesis is supported by several analyses of GI networks. In the yeast GI network a path length of two is the best measure of relationships between protein and GIs [26]. Moreover, analysis of the yeast GI network shows a characteristic path length of 3.3, suggesting a high density of GI interactions [27]. The GI node filter recovered more than double the true positives as expected; however, we observed a ∼4 fold increase in the number of true negatives. This filter has been made available on the MnM website, as it has the advantage of having a higher sensitivity.

Beyond prediction of minimotifs, the GI filter provides a tool to examine GIs at a finer level of granularity. Identification of a GI infers that two genes have a related function in a complementary pathway or in the same pathway. However, genetics does not identify how the two genes are related. Protein-protein interaction networks can help to identify the relationships, but only a fraction of GIs have known protein-protein interactions. This could be due to the fact that different protein-protein interaction databases do not yet have extensive redundancy, suggesting that there are many protein-protein interactions yet to be discovered. However, if protein-protein interactions are transient, such as in an enzyme/substrate relationship or in a highly regulated signaling complex, these interactions are not likely to be identified in a protein-protein interactions screen, but may be detected in a GI screen. In support of this contention, 56% of minimotifs with a posttranslational modification activity/substrate relationship had a known GI, whereas only 19% of minimotifs with a binding activity had a GI. This is the particular case where our new GI tool will help to identify binding, trafficking and enzymatic functions for known GIs. The user, simply enters the query source protein, identify s of a pair of genetically interacting proteins and looks for a relationship with the partner protein. Furthermore, this tool is also likely to assist in construction of pathways in a similar manner.
